# Synergistic Electronic Modulation in Nitrogen, Sulfur, and Boron-Doped Graphene Nanoribbons for Enhanced Oxygen Reduction Electrocatalysis

**DOI:** 10.1021/acsomega.5c12983

**Published:** 2026-02-24

**Authors:** Giancarlo S. Dias, Matthew Labbe, Anqiang He, Richard Landers, Josiel M. Costa, Ambrósio F. de Almeida Neto, Douglas G. Ivey

**Affiliations:** † Departamento de Desenvolvimento de Processos e Produtos, Faculdade de Engenharia Química (FEQ), 28132 Universidade Estadual de Campinas (UNICAMP), Avenida Albert Einstein, 500, 13083-852 Campinas, São Paulo Brazil; ‡ Department of Chemical and Materials Engineering, 3158 University of Alberta, Edmonton, Alberta T6G 1H9, Canada; § Departamento de Física Aplicada, Instituto de Física GLEB Wataghin (IFGW), Universidade Estadual de Campinas (UNICAMP), Rua Sérgio Buarque de Holanda, 777, 13083-859 Campinas, São Paulo Brazil; ∥ Faculdade de Engenharia de Alimentos (FEA), Universidade Estadual de Campinas (UNICAMP), Rua Monteiro Lobato, 80, 13083-862 Campinas, São Paulo Brazil

## Abstract

The sluggish kinetics of the oxygen reduction reaction (ORR) remains one of the main challenges in the development of efficient and sustainable metal-free catalysts for energy conversion and storage devices. Multielement doping of carbon materials has emerged as an effective strategy to tailor their electronic properties and enhance ORR activity. In this study, graphene nanoribbons codoped with nitrogen, sulfur, and boron (NSB-GNR) were prepared via a facile hydrothermal route, comprehensively characterized and evaluated for ORR catalysis. Characterization by EELS, FTIR, XPS, and ICP-OES confirmed successful heteroatom incorporation and revealed that boron was mainly located in the inner layers of NSB-GNR. Raman analysis suggested that boron incorporation may have induced defect reconstruction within the carbon lattice. Nitrogen adsorption–desorption and zeta potential analyses indicated that the acidic environment generated by boric acid during hydrothermal synthesis partially neutralized surface charges, leading to reduced BET surface area (176 m^2^ g^–1^) and pore volume (0.23 cm^3^ g^–1^) compared with the N,S-doped counterpart. Despite this reduction, NSB-GNR exhibited superior ORR performance with an onset potential of 0.805 V, half-wave potential of 0.658 V vs RHE, and a limiting current density of −3.24 mA cm^–2^, following an efficient four-electron transfer pathway. These findings demonstrate that the synergistic interactions among nitrogen, sulfur, and boron dominate over textural effects, providing new insights into the cooperative electronic modulation of heteroatoms and offering a scalable strategy for designing advanced metal-free carbon electrocatalysts.

## Graphical abstract

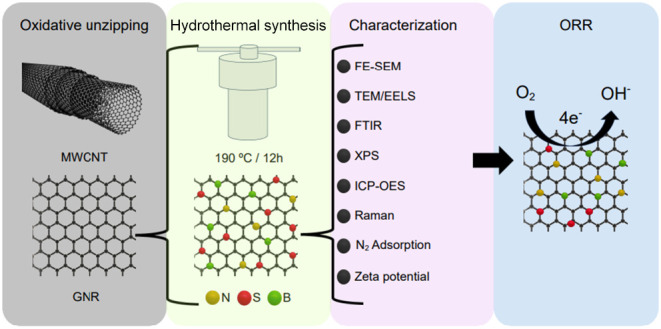

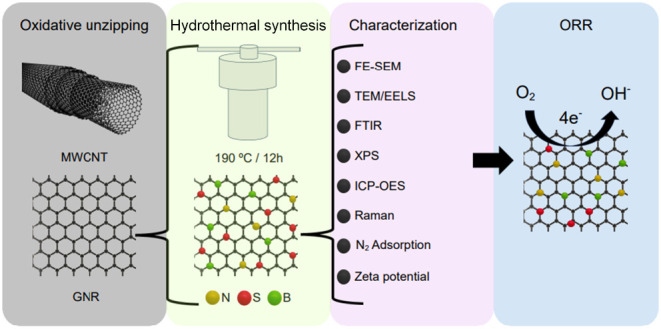

## 1 Introduction

The oxygen reduction reaction (ORR) is a fundamental reaction in electrochemistry for energy conversion in fuel cells and metal-air batteries.^1^ In these devices, oxygen is reduced at the air electrode via two possible pathways depending on the adsorption configuration of the oxygen molecule. In the desired four-electron (4e^–^) pathway, the parallel adsorption of two O atoms favors O_2_ dissociation, leading to the direct reduction of O_2_ to hydroxide ions (OH^–^). In contrast, perpendicular O_2_ adsorption favors the undesired sequential 2 × 2e^–^ pathway, generating peroxide, which lowers the overall efficiency.^2–4^ Although these reactions are thermodynamically feasible, the activation and dissociation of O are kinetically hindered due to the strong O=O bond energy (498 kJ mol^–1^), requiring the use of electrocatalysts to lower the energy barrier and facilitate bond cleavage.^5,6^ Platinum supported on carbon black (Pt/C) is recognized as the benchmark catalyst for ORR. However, its high cost and limited operational stability restrict large-scale implementation.^7^ Consequently, efforts have focused on developing affordable, earth-abundant catalysts with comparable performance.

Metal-free carbon materials have emerged as promising electrocatalysts to replace Pt/C due to their low cost, high stability in alkaline environments, large surface areas, good conductivity, and tunable electronic structure.^8^ Among them, graphene nanoribbons (GNRs), quasi-one-dimensional strips of graphene, exhibit good electrical properties arising from a lateral quantum confinement effect and abundant edge defects.^9^ In particular, zigzag edges have been identified as active sites for the ORR.^10^ To further enhance the catalytic performance, heteroatoms are commonly incorporated into the GNR lattice to disrupt the sp^2^ carbon framework and modulate the π-electron distribution.^11^ Nitrogen is the most frequently employed heteroatom due to its strong electronegativity and diverse configurations that effectively improve the ORR through distinct electronic effects.^12–14^ In addition to nitrogen, sulfur and boron have also been introduced as dopants. Boron introduces electron-deficient (p-type) sites that favor oxygen adsorption,^15^ whereas sulfur primarily enhances catalytic performance by inducing lattice distortions through its larger atomic radius.^16^ Building on these effects, dual-doped GNRs have been explored to take advantage of the synergistic interaction between different heteroatoms.^17–19^ Indeed, it was reported that sulfur can change the charge and spin density of neighboring nitrogen atoms, further facilitating O_2_ adsorption.^20,21^ Furthermore, N–B bonding can improve the ORR performance by increasing electrode wettability and facilitating oxygen diffusion kinetics.^22–24^ Given the beneficial synergistic effects observed in dual-doped catalysts, research has recently shifted toward tertiary-doped carbon materials incorporating nitrogen, sulfur, and boron. The first attempt to explore such triple-doped catalysts for ORR catalysis was reported by Liu et al.,^25^ who investigated N, S, and B codoped carbon nanotubes. More recently, Chauhan et al.^26^ employed waste diesel soot as a carbon matrix, while Xiao et al.^27^ developed lignin-derived N–S–B codoped carbon catalysts. These studies demonstrated superior ORR activity of the tridoped catalysts compared with their dual-doped counterparts, highlighting the potential of multiheteroatom synergy. However, the fundamental understanding of the complex interplay among nitrogen, sulfur, and boron in the ORR process remains limited. Moreover, these studies used complex synthesis routes, which required very high temperatures (700 °C – 900 °C) and inert or vacuum environments, making them costly and difficult to reproduce.

Herein, we aim to provide new insights into the synergistic effects of N, S, and B codoping on the ORR mechanism and to propose a simpler synthesis route. To this end, N, S, and B codoped graphene nanoribbons (NSB-GNR) were prepared via a facile, low-temperature hydrothermal method, offering a sustainable and scalable alternative for the design of metal-free ORR catalysts.

## 2 Materials and Methods

### 2.1 Chemicals

Multiwalled carbon nanotubes (MWCNT) were purchased from MKnano (Canada). Urea, potassium hydroxide, potassium permanganate, sodium sulfide hydrate, and reagent alcohol (ethanol) were purchased from Thermo Fisher Scientific. Thiourea and Pt, nominally 40% on high surface area advanced carbon supports, were obtained from Alfa Aesar. Boric acid was obtained from MP Biomedicals. Sulfuric acid (95.0–98.0% w/w) was obtained from LabChem. Perfluorosulfonic acid (PFSA) dispersion (D5, 5%) was purchased from the FuelCell Store. Sodium nitrate was acquired from Sigma-Aldrich. Hydrogen peroxide was purchased from NEON (Brazil). Nitric acid (65.0% w/w) was acquired from Labsynth (Brazil). All chemicals were of analytical grade.

### 2.2 Preparation of Multidoped GNR Electrocatalysts

Catalysts were synthesized through a one-step hydrothermal process. Initially, as-received MWCNTs were longitudinally unzipped using a modified Hummers’ method to obtain oxidized GNRs.^28^ Subsequently, 140 mg of oxidized GNRs, 0.5 g of urea, 0.5 g of thiourea, and 0.5 g of boric acid were added to 70 mL of deionized (DI) water and sonicated for 45 min. The resulting suspension was transferred into a 100 mL Teflon-lined stainless-steel autoclave and treated hydrothermally at 190 °C for 12 h. After cooling to room temperature, the catalyst was collected by centrifugation (10,000 rpm for 15 min), washed thoroughly with DI water and ethanol, and dried at 60 °C overnight. The final product was labeled as NSB-GNR. For comparison, dual-doped GNRs were obtained by the same synthesis route, with the precursors adjusted to urea + thiourea for NS-GNR and urea + boric acid for NB-GNR.

### 2.3 Physicochemical Characterization

The surface morphology of the catalysts was analyzed by field-emission scanning electron microscopy (FE-SEM, Zeiss Sigma 300 VP) and by transmission electron microscopy (TEM, JEOL JEM-2100F) equipped with an electron energy-loss spectroscopy (EELS) analyzer. Chemical structures and bonding characteristics were identified through Fourier transform infrared spectroscopy (FTIR, Nicolet 6700, Thermo Scientific) in the spectral range of 650–4000 cm^–1^. Surface composition and chemical bond characteristics were examined by X-ray photoelectron spectroscopy (XPS, Kratos Axis spectrometer, monochromatized Al Kα source, 1486.69 eV). The structural ordering and degree of graphitization of the catalysts were assessed by *in situ* Raman spectroscopy in the range of 200–2500 cm^–1^ (Renishaw inVia Raman microscope). The textural properties were characterized by nitrogen adsorption–desorption isotherms (Quantachrome Autosorb-iQ gas adsorption analyzer). Specific surface area and pore size distribution were determined by the Brunauer–Emmett–Teller (BET) and Barrett–Joyner–Halenda (BJH) methods, respectively. Zeta-potential (ξ) and electrophoretic mobility (EM) were measured over a pH range of 2–12 (Malvern Zetasizer Nano ZSP). At pH 13, the dispersion was highly unstable, leading to rapid particle sedimentation. The pH values of colloidal suspensions (1.0 mg mL^–1^) were adjusted by adding 0.1 M sulfuric acid or 0.1 M potassium hydroxide.

Inductively coupled plasma optical emission spectroscopy (ICP-OES, Thermo Fisher Scientific iCAP 6300 Duo Series) was employed to quantify the boron and sulfur contents in NSB-GNR, NB-GNR, NS-GNR, and pristine MWCNT (used as a control). The monitored emission lines were 249 nm for boron and 182 nm for sulfur. The ICP-OES operating conditions included a plasma power of 1350 W, a nebulizer gas flow of 0.80 L min^–1^, and an auxiliary gas flow of 0.50 L min^–1^. A concentric nebulizer coupled with a cyclonic spray chamber was used as the sample introduction system. The limits of detection (LOD) and quantification (LOQ) were determined in accordance with IUPAC recommendations, using the standard deviation of replicate analytical blanks: LOD = 3σ_blank/*m* and LOQ = 10σ_blank/*m*, where *m* is the slope of the calibration curve. Prior to ICP-OES analysis, the samples underwent dry washing at 650 °C for 4 h, followed by wet acid digestion in a mixture of concentrated nitric acid (3 mL) and hydrogen peroxide (30%, 1 mL) at 120 °C for 8 h.^29^

### 2.4 Electrochemical Measurements

ORR electrochemical measurements were performed at room temperature (25 °C) with a BioLogic VSP potentiostat (BioLogic Science Instruments) in a conventional three-electrode configuration using oxygen-saturated 0.1 M potassium hydroxide as the electrolyte. A glassy carbon rotating disk electrode (GCE, 5.0 mm diameter) was employed as the working electrode, a platinum coil as the counter electrode, and a Hg/HgO electrode as the reference electrode. The multidoped GNR and commercial Pt/C inks were prepared by mixing 4 mg of catalyst with 0.25 mL PFSA solution and 0.75 mL ethanol followed by sonication in an ice bath for 1 h. Then, 60 μL of the ink was drop-cast onto the GCE and dried under a 20 W heat lamp for 1 h, resulting in a mass loading of ∼0.1 mg cm^–2^. Prior to ORR measurements, the as-prepared working electrode was electrochemically activated by cyclic voltammetry (CV) in the potential range from 0.2 to −0.7 V vs Hg/HgO at a scan rate of 20 mV s^–1^ until stable and reproducible CV curves were obtained.

Linear sweep voltammetry (LSV) was recorded at rotation speeds of 100, 400, 900, and 1600 rpm, sweeping the potential from 0.2 to −0.7 V vs Hg/HgO. The catalytic kinetics were evaluated by determining the Tafel slope and the electron transfer number. The Tafel slope was obtained according to (eq 1), where η is the overpotential, *a* is the intercept, and *b* is the Tafel slope.

1 $$\eta =a+b\log J_{K}$$

The kinetic current (*J*_K_) was calculated from the LSV curve at 1600 rpm after correcting for mass transport effects using (eq 2), where *J* and *J*_L_ are the measured and limiting current densities, respectively.^30^

2 $$J_{K}=\frac{J\times J_{L}}{J_{L}-J}$$

The number of electrons transferred (*n*) was determined from LSV curves recorded at rotation speeds of 100–1600 rpm using the Koutecký–Levich (K–L) eq (eq 3), where ω is the electrode angular velocity, and *B* is the Levich constant, defined in (eq 4).^27^

3 $$\frac{1}{J}=\frac{1}{J_{L}}+\frac{1}{J_{K}}=\frac{1}{B\omega^{1/2}}+\frac{1}{J_{K}}$$

4 $$B=0.62\mathrm{nF}{\left(D_{0}\right)}^{2/3}{\left(v\right)}^{-1/6}C_{0}$$

In eq 5, *F* is the Faraday constant (96,485 C mol^–1^), *D*_0_ is the diffusion coefficient of oxygen in 0.1 M potassium hydroxide (1.9 × 10^–5^ cm^2^ s^–1^), *C*_0_ is the bulk concentration of oxygen (1.2 × 10^–6^ mol cm^–3^), and ν is the kinematic viscosity of the electrolyte (1 × 10^–2^ cm^2^ s^–1^). All electrochemical measurements were IR-compensated with respect to the Hg/HgO reference electrode and performed in triplicate to ensure repeatability.

Long-term ORR stability was evaluated by chronoamperometry at – 0.210 V vs Hg/HgO for 85 000 s. The NSB-GNR working electrode was prepared by spray-coating the catalyst ink onto a gas diffusion layer and tested in O_2_-saturated 1.0 M KOH. The ink was prepared by dispersing 10 mg of catalyst in 200 μL isopropanol, 400 μL deionized water, and 20 μL PFSA solution, followed by sonication in an ice bath for 1 h and drying at room temperature.

All potentials were converted to RHE using eq 5.^31^

5 $$E_{\mathrm{RHE}}=E_{\mathrm{Hg}/\mathrm{HgO}}+0.0591\times \mathrm{pH}+E_{\mathrm{Hg}/\mathrm{HgO}}^{0}$$

where *E*_Hg/HgO_ is the measured potential and *E*_Hg/HgO_^0^ is the standard potential of the Hg/HgO electrode (0.098 V vs RHE).

## 3 Results and Discussion

### 3.1 Preparation and Physicochemical Characterization of NSB-GNR

Figure 1a illustrates the schematic synthesis route of NSB-GNRs. The as-received MWCNTs (Figure 1b) were chemically unzipped through oxidative treatment, yielding multilayer GNRs with a typical quasi-one-dimensional morphology (Figure 1c).^28,32^ The proposed unzipping mechanism suggests that the oxidative process generates a high density of edge defects and introduces oxygen functionalities (carboxyl, carbonyl, hydroxyl and epoxide) into the carbon framework.^33,34^ Such defects and functional groups are known to act as anchoring sites for heteroatom incorporation.^35,36^ Indeed, the EELS mapping confirms the homogeneous distribution of nitrogen, boron, and sulfur heteroatoms throughout the GNR framework after the one-step hydrothermal treatment (Figure 1e–j).

**Figure 1:**
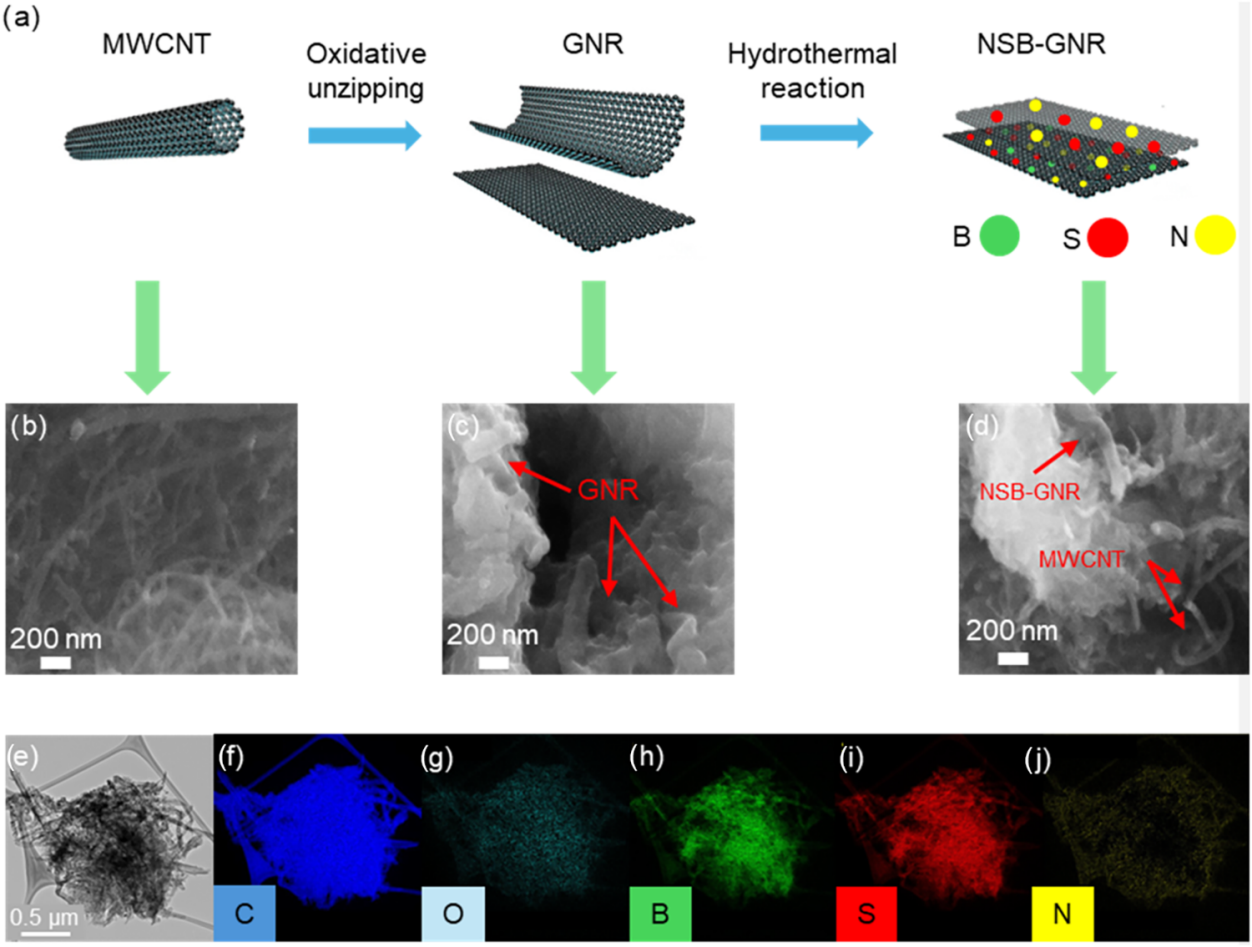
(a) Schematic synthesis route for NSB-GNRs; FE-SEM secondary electron (SE) images of (b) MWCNTs, (c) GNRs, and (d) NSB-GNRs; (e) TEM bright field image of NSB-GNRs; (f–j) carbon, oxygen, boron, sulfur, and nitrogen EELS elemental mapping of the area in (e).

The FE-SEM image of NSB-GNRs (Figure 1d) shows that the GNR morphology was well preserved during hydrothermal synthesis. Interestingly, Figure 1d also reveals a few residual MWCNTs in the sample. Kosynkin et al.^33^ demonstrated that increasing the concentration of potassium permanganate leads to a progressive decrease in nanotube diameter, indicating that optimized oxidant concentration is crucial for achieving complete unzipping of all concentric graphene walls. Considering that the same oxidation conditions were adopted as those reported by Chen et al.,^28^ who observed no partially unzipped nanotubes, the presence of residual MWCNTs in the sample may be attributed to the inherent heterogeneity of diameter and length of the MWCNTs. Furthermore, morphological analysis shows that both NSB-GNRs (Figure 1d) and NB-GNRs (Figure 2a) exhibit a similar, enhanced degree of clustering when compared with NS-GNRs (Figure 2b). This increased aggregation is likely due to the addition of boric acid during hydrothermal synthesis, which lowered the suspension pH from 8.1 to 5.3, thereby partially neutralizing surface charges, as will be discussed in detail later.

**Figure 2:**
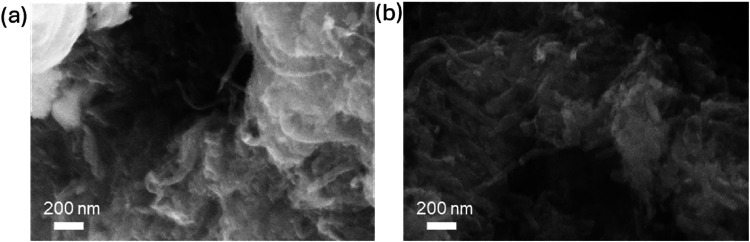
FE-SEM SE images of (a) NB-GNR and (b) NS-GNR catalysts.

The bonding modes of the synthesized catalysts were evaluated by FTIR. As shown in Figure 3a, the spectra for NSB-GNR, NB-GNR, and NS-GNR share characteristic absorption bands at 3418, 2923, 2853, 2360, 1733, 1563, 1455, and 1177 cm^–1^, which are assigned to −OH and –NH stretching vibrations, symmetric and asymmetric stretching of methylene, atmospheric CO_2_, carbonyl stretching of the carboxylic group, C=C stretching, C–N stretching in amide groups, and C–O stretching, respectively.^37–39^ The C–N and N–H vibrations in all three samples confirm the successful incorporation of nitrogen into the carbon framework. In the NS-GNR spectrum, characteristic C–S vibrations appear at ∼750 cm^–1^,^40^ while a broad band with a peak around 1000 cm^–1^ can be attributed to contributions from S=O stretch, sulfonic esters vibrations, and symmetric sulfonic acid groups.^39–41^ In the NB-GNR spectrum, additional bands are observed at 3220, 1396, and 822 cm^–1^, corresponding to B–OH stretching, in-plane B–N transverse stretching, and out-of-plane B–N–B tensile vibration, respectively. Furthermore, the bands at 1228, 1100, and 1026 cm^–1^ can be assigned to B–C vibrations.^42–44^ The NSB-GNR spectrum exhibits broad bands in the 825–1287 and 1340–1600 cm^–1^ regions. These bands are consistent with the overlapping boron- and sulfur-related vibrations observed in NB-GNR and NS-GNR spectra, confirming the successful incorporation of boron and sulfur in the carbon matrix. Additionally, in the NSB-GNR spectrum, the B–OH and B–N–B bands are absent or at least broadened, suggesting that the sulfur incorporation into the carbon matrix modifies local bonding interactions, potentially competing for anchoring sites and affecting the structural arrangement of neighboring B- and N-containing groups.

**Figure 3:**
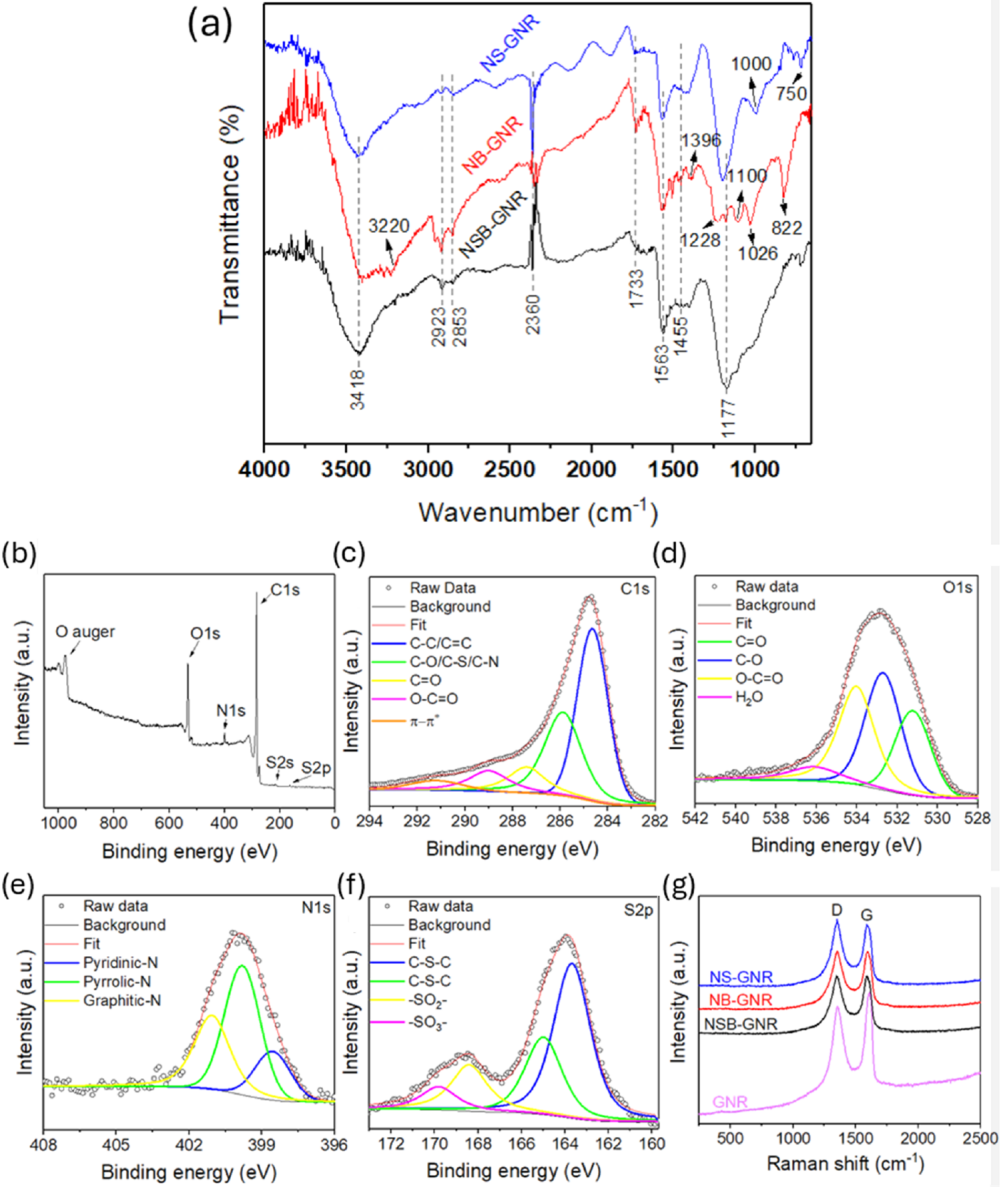
(a) FTIR spectra for NSB-GNR, NB-GNR, and NS-GNR; (b) XPS survey spectrum and high-resolution spectra for (c) C 1s, (d) O 1s, (e) N 1s and (f) S 2p of the NSB-GNR catalyst; (g) Raman spectra for NSB-GNR, NB-GNR, NS-GNR, and GNR.

XPS analysis was performed to investigate the surface composition and chemical states of NSB-GNR. Notably, both the XPS survey spectrum (Figure 3b) and the high-resolution B 1s spectrum (Figure S1) reveal no evidence of boron on the catalyst surface. A similar result is observed for NB-GNR (Figure S2a). Given that XPS is a surface-sensitive technique with a typical probing depth of ∼2–10 nm, and that FTIR and EELS analyses indicated the presence of boron, it is reasonable to infer that boron is embedded in the inner catalyst layers.^45,46^ To verify this hypothesis, ICP-OES was performed, confirming the presence of boron throughout the bulk of both NSB-GNR and NB-GNR catalysts. The boron content in NSB-GNR is 1.6 times higher than that in NB-GNR (Table 1).

**Table 1:** Elemental Concentration of Boron and Sulfur in NSB-GNR, NB-GNR, NS-GNR, and Pristine CNT (Control) Determined by ICP-OES

samples	*B* (mg g_cat_^–1^)	*S* (mg g_cat_^–1^)
NSB-GNR	10.06	37.40
NB-GNR	6.55	2.14
NS-GNR	n.d.	24.22
pristine CNT	n.d.	14.91

The XPS survey spectrum for NSB-GNR (Figure 3b) displays characteristic peaks of C, O, N, and S, confirming the successful incorporation of nitrogen and sulfur into the catalyst surface. The C 1s spectrum (Figure 3c) was deconvoluted into five peaks, assigned to C–C/C=C (284.6 eV), C–S/C–N/C–O (285.8 eV), C=O (287.3), O–C=O (288.9 eV), and the π–π* shakeup satellite (291.2 eV).^19,47–49^ The O 1s spectrum shows four distinct peaks (Figure 3d). The three peaks at 531.2, 532.6, and 533.9 eV are ascribed to carbonyl, epoxy, and carboxyl oxygen functional groups, respectively.^50,51^ The peak at 536.1 eV corresponds to adsorbed water from the atmosphere.^52^ The C 1s and O 1s high-resolution spectra for NB-GNR (Figure S2b,c, respectively) and NS-GNR (Figure S3b,c, respectively) were deconvoluted in a similar manner.

The N 1s spectrum for NSB-GNR (Figure 3e) shows pyridinic-N (398.6 eV), pyrrolic-N (399.8 eV), and graphitic-N (401.1 eV). A similar deconvolution was conducted for the N 1s spectra of NB-GNR (Figure S2d) and NS-GNR (Figure S3d).^53^ Pyrrolic-N is the predominant nitrogen species, accounting for ∼48% of the total nitrogen, a trend consistent with NB-GNR and NS-GNR (Table 2). The predominance of pyrrolic-N has been widely reported in various carbon matrices.^25–27^ Notably, the data presented in Table 2 suggest that boron may influence the relative distribution of pyrrolic-N and graphitic-N species within the GNR framework. Although pyrrolic-N is often reported to favor the ORR via a two-electron pathway, the specific contribution of each nitrogen configuration to the overall ORR activity remains a subject of debate.^54^ While some studies highlight the role of pyridinic-N and pyrrolic-N in enhancing the ORR, others suggest graphitic-N as the most active species.^55^ A recent study reported superior ORR performance for a catalyst enriched in pyrrolic-N and graphitic-N.^56^

**Table 2:** Surface Elemental Composition (Relative to the Total Amount of Carbon) and Deconvoluted XPS Data for N and S Species in NS-GNR, NB-GNR, and NSB-GNR

element/species	NS-GNR	NB-GNR	NSB-GNR
O/C	0.175	0.202	0.173
S/C	0.011	n.d.	0.009
N/C	0.047	0.068	0.027
N Species (atom %)			
pyridinic-N	1.08 (20.4^a^)	1.37 (19.3^a^)	0.57 (18.2^a^)
pyrrolic-N	3.02 (57.1)	2.90 (40.7)	1.51 (48.0)
graphitic-N	1.19 (22.5)	2.29 (32.1)	1.07 (33.8)
oxidized-N	n.d.	0.56 (7.9)	n.d.
S Species (atom %)			
C–S–C	0.50 (72.4^a^)	n.d.	0.45 (75.4^a^)
C–SO_*x*_-C	0.19 (27.6)	n.d.	0.15 (24.6)

a Represents relative amount.

The S 2p spectrum for NSB-GNR was deconvoluted into four peaks (Figure 3f), located at 163.7, 165.0, 168.4, and 169.8 eV, which correspond to C–S–C 2p_3/2_, C–S–C 2p_1/2_, −SO_2_−, and −SO_3_− species, respectively.^57,58^ A similar deconvolution was performed for the S 2p spectra of NS-GNR (Figure S3e). The thiophene-like species (C–S–C) changes the spin and charge densities of adjacent carbon atoms, which is associated with improved ORR performance.^59^ Although oxidized sulfur species are often reported as catalytically inactive for ORR, Maiti et al.^60^ demonstrated, both experimentally and computationally, that edge-located oxidized sulfur dopants in GNRs can modify the spin density of neighboring carbon atoms, reducing the overpotential and promoting the 4e^–^ reduction pathway. Regardless, in NSB-GNR and NS-GNR catalysts, sulfur is predominantly present as thiophene-like species, with a relative amount of ∼75% (Table 2).

The degree of graphitization and the presence of structural defects in the carbon-based catalysts were investigated by Raman spectroscopy (Figure 3g). The catalyst spectra reveal two characteristic peaks at ∼1350 cm^–1^ (D band, corresponding to the vibrations of disordered sp^3^-bonded carbon atoms) and ∼1595 cm^–1^ (G band, corresponding to the vibrations of graphitic sp^2^-bonded carbon atoms).^61^ As the D band is activated by any breaking of symmetry of the carbon lattice, such as sp^3^-type defects, vacancy sites, edge defects, heteroatom incorporation, and Stone–Wales topological defects, it is not suitable for direct characterization of doping. Instead, the G band is more sensitive to doping-induced electronic perturbations.^62^ As shown in Figure 3g, heteroatom incorporation caused the G-band position of GNR to shift from 1610 to 1592 cm^–1^, accompanied by an increase in its full width at half-maximum (fwhm) from 62 to 74 cm^–1^, confirming doping induced electronic perturbations.^62^ The downshift of the G band and the broadening observed for the doped GNR catalysts suggest a reduction in charge carrier concentration, likely associated with the partial removal of oxygen-containing functional groups.^11,63^ Beyond electronic modulation, heteroatom incorporation can modify the carbon lattice by inducing local structural perturbations, including lattice distortion and partial sp^2^ → sp^3^ rehybridization, which are beneficial for oxygen electrocatalysis.^64,65^ These extrinsic perturbations are manifested as an enhanced D-band intensity in Raman spectra. Accordingly, the intensity ratio of the D and G bands (*I*_D_/*I*_G_) increased from 0.86 for pristine GNR to values exceeding 1.0 for the doped catalysts. Among them, NS-GNR exhibited the highest *I*_D_/*I*_G_ ratio (1.11), reflecting a higher degree of local structural disorder primarily associated with sulfur incorporation, which has a larger atomic radius than carbon and longer C–S bond length. In this context, a higher *I*_D_/*I*_G_ ratio would be expected for NSB-GNR due to its higher sulfur content, as evidenced by ICP–OES analysis (Table 1). However, NSB-GNR exhibited an *I*_D_/*I*_G_ ratio of 1.05, comparable to that of NB-GNR (1.05). This reduction may be associated with the catalytic role of boron in promoting partial graphitization or defect reconstruction in graphene-based materials, as reported in recent studies.^66,67^

Heteroatom doping influences the electrical conductivity of graphene nanoribbons by modifying their electronic structure and defect density.^68^ Nitrogen dopants, particularly in graphitic configurations, can enhance charge carrier density, whereas pyrrolic-N and thiophene-like sulfur introduce localized states and lattice distortions that may reduce carrier mobility. Boron acts as a p-type dopant, lowering the local Fermi level and further modulating charge transport. In this study, Raman G-band shifts and broadening indicate doping induced electronic perturbations, while the preserved sp^2^ carbon framework suggests that sufficient electrical conductivity is maintained. The high limiting current densities and stable ORR kinetics (discussed in Section 3.2) further indicate that charge transport is not rate limiting under the investigated conditions.

The surface area and pore structure of NSB-GNR, NB-GNR, and NS-GNR were analyzed by nitrogen physisorption. As shown in Figure 4a, the isotherms can be classified as a combination of IUPAC Types II and IV with an H3-type hysteresis loop, characteristic of mesoporous materials containing narrow fissure-type pores.^69^ This is consistent with the stacked, sheet-like morphology of the GNRs (Figure 1c), where the interlayer space forms slit-like pores. Figure 4b shows that all catalysts exhibit a unimodal pore size distribution. The BET surface area, pore volume, and pore width are listed in Table 3. The pore diameter of ∼3.83 nm for all samples confirms the mesoporous structure of the catalysts. Such mesoporosity is beneficial for ORR, as it combines high surface area with improved electrolyte diffusion, thus developing the essential triple-phase boundary for ORR. NS-GNR exhibits the highest pore volume and surface area, followed by NSB-GNR and NB-GNR. This is consistent with the agglomeration of the GNR sheets, likely induced by partial neutralization of surface charges upon the addition of boric acid during catalyst synthesis. Sheet agglomeration reduces the interlayer spacing, thereby limiting the accessible surface area.

**Figure 4:**
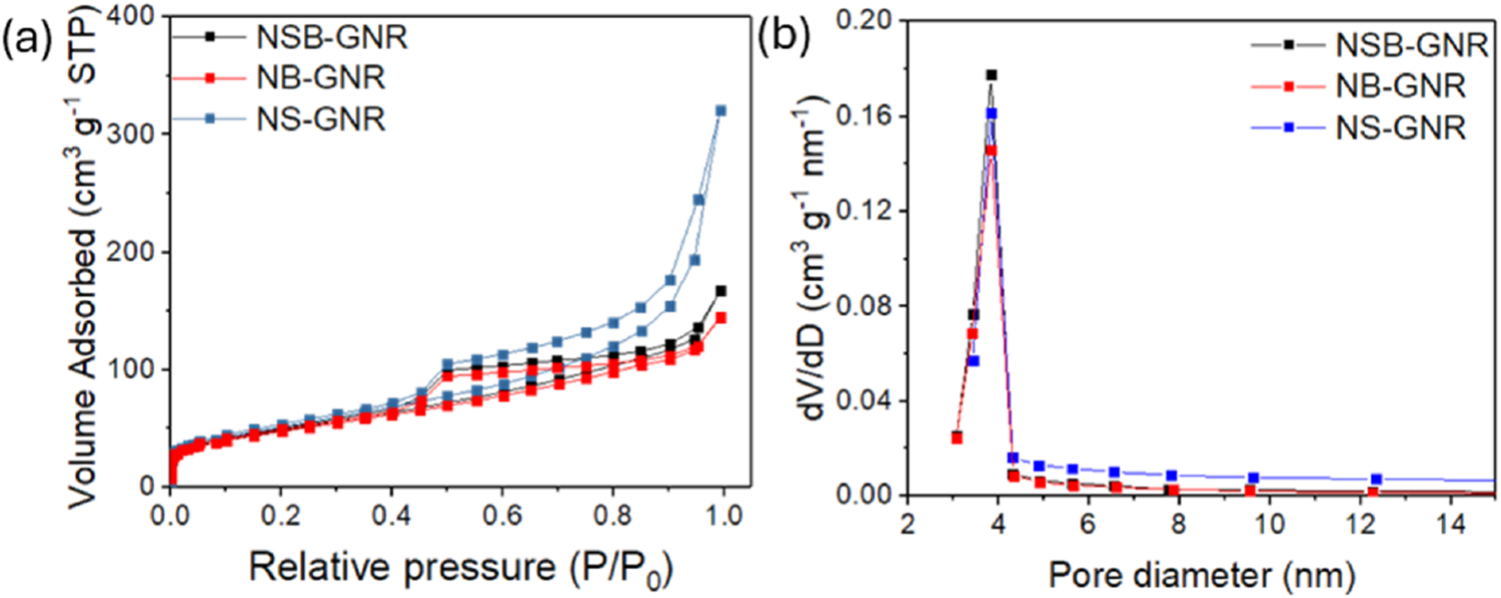
(a) Nitrogen adsorption–desorption isotherms and (b) pore size distribution of NSB-GNR, NB-GNR, and NS-GNR.

**Table 3:** Textural Characteristics of NSB-GNR, NB-GNR, and NS-GNR Catalysts

sample	*S*_BET_ (m^2^g^–1^)	*V*_pore_ (cm^3^g^–1^)	*D*_pore width_ (nm)
NSB-GNR	176.4	0.232	3.82
NB-GNR	172.1	0.194	3.83
NS-GNR	187.8	0.476	3.83

The ζ-potential represents the electrostatic potential at the slipping plane within the electrical double layer relative to the bulk electrolyte. It is widely used as an indicator of electrostatic stabilization, with absolute values exceeding 30 mV generally considered sufficient to prevent aggregation.^70,71^ To evaluate stability, the ζ-potentials of NSB-GNR, NB-GNR, and NS-GNR were measured as a function of pH. As shown in Figure 5a, all catalysts exhibit an isoelectric point (IEP) near pH 2.6 (ζ ≈ 0). Beyond the IEP, their absolute ζ-potentials increase with pH, with the largest value at pH 12. At this pH, the ζ-potential values for NSB-GNR, NB-GNR, and NS-GNR are −38.5, −37.7, and −40.1 mV, respectively, clearly indicating highly stable colloidal suspensions composed of negatively charged nanoparticles (ζ < 0). This pH dependence arises because the addition of hydroxide ions promotes the deprotonation of surface hydroxyl groups. The resulting negatively charged oxygen species confer a net negative charge to the particle surface. Consequently, the increase in surface charge density leads to stronger electrostatic repulsion between particles and, thus, a higher absolute zeta potential.^72^

**Figure 5:**
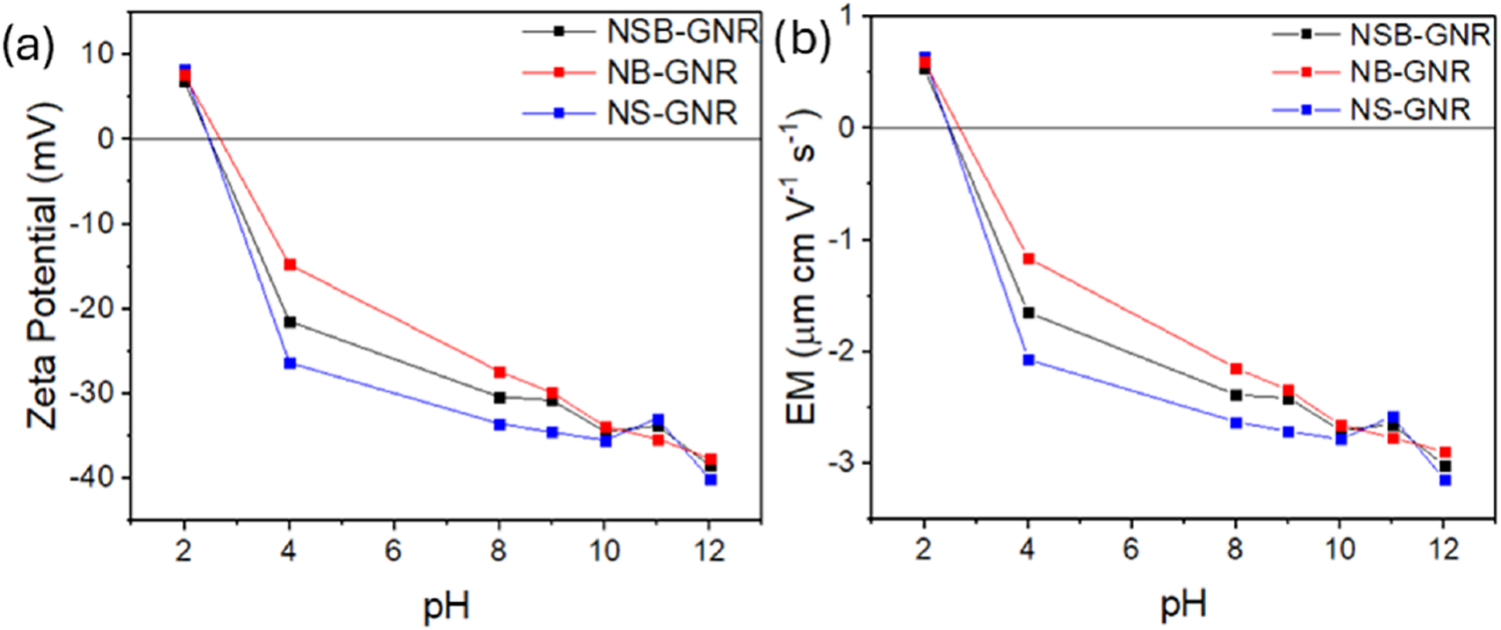
(a) ζ-potential and (b) EM as a function of pH for NSB-GNR, NB-GNR, and NS-GNR.

The ζ-potential measurements can further corroborate the earlier observations of a higher degree of agglomeration for NSB-GNR and NB-GNR relative to NS-GNR, as shown in the SEM (see Figures 1d and 2a,b) and BET (see Table 3) results. As previously mentioned, the addition of boric acid during the synthesis of NSB-GNR and NB-GNR lowered the pH of the suspension to 5.3, down from 8.1 for NS-GNR. This reduction in pH potentially leads to a reduction in the number of surface charges and the promotion of particle agglomeration. At the synthesis pH of 8.1, NS-GNR exhibits a ζ-potential with a magnitude of 33.5 mV, which is higher than the stability threshold (30 mV). For NSB-GNR and NB-GNR, the magnitude of the ζ-potential decreases with decreasing pH (Figure 5a), remaining below the stability threshold of 30 mV at low pH levels, including at the synthesis pH of 5.3. This decrease in the magnitude of the ζ-potential for the boron-doped catalysts suggests a reduction in electrostatic repulsion, leading to particle agglomeration, consistent with SEM and BET observations. Moreover, the lower magnitude ζ-potentials for NB-GNR compared with the other two catalysts over the range of mild pH values is also consistent with NB-GNR exhibiting the lowest BET surface area and pore volume (Table 3). This behavior for NB-GNR is likely the result of a smaller number of ionizable surface groups.

The EM determines how fast a particle moves under the influence of an electric field. According to the Smoluchowski equation, for a given solvent and under fixed operational conditions (e.g., temperature), the EM depends solely on the ζ-potential.^73^ This is reflected by the similar profiles for the ζ-potential (Figure 5a) and EM (Figure 5b) plots in Figure 5. At pH 12, the EM values for NSB-GNR, NB-GNR, and NS-GNR are −3.0, −2.9, and −3.1 μm cm V^–1^ s^–1^, respectively. These values are in line with those previously reported for carbon materials.^74,75^

### 3.2 ORR Electrochemical Performance

In heteroatom-doped graphene nanoribbons, the ORR activity is generally associated with carbon atoms adjacent to heteroatom dopants rather than the heteroatoms acting as isolated active centers.^11^ The incorporation of N, S, and B perturbs the local charge and spin density of neighboring carbon atoms, particularly at edge and defect-rich regions, which are abundant in graphene nanoribbons. These electronically activated carbon sites are, therefore, considered the true active sites for ORR in metal-free GNR-based catalysts.

Figure 6a displays the LSV curves for NSB-GNR, NB-GNR, and NS-GNR. NSB-GNR clearly exhibits superior ORR catalytic activity, with a higher limiting current density and more positive onset (*E*_onset_, measured at 0.1 mA cm^–2^) and half-wave (*E*_1/2_) potentials (Table 4). Furthermore, as shown in Figure 6b, NSB-GNR follows a four-electron transfer pathway, whereas the intermediate electron transfer numbers of NB-GNR and NS-GNR, between 2 and 4, indicate a sequential 2 × 2e^–^ pathway with peroxide formation. The Tafel slope provides important information on the rate-determining step (RDS) of the ORR. Typically, a slope of about 120 mV dec^–1^ reflects a process limited by the initial electron transfer or by the first surface reaction occurring under high coverage of oxygenated intermediates.^76,77^ When the slope decreases to around 60 mV dec^–1^, the kinetics are governed by a subsequent chemical transformation involving the adsorbed intermediates. In this case, the dependence of current on potential mainly arises from changes in the surface coverage of reactive species.^77,78^ As depicted in Figure 6c, the NS-GNR catalyst shows a Tafel slope near 60 mV dec^–1^, indicating that its ORR kinetics are governed by a chemical step associated with the adsorbed intermediates. In contrast, both NB-GNR and NSB-GNR exhibit slopes close to 120 mV dec^–1^, suggesting that boron incorporation shifts the RDS toward an electron-transfer-controlled mechanism. Boron doping, especially in the presence of nitrogen atoms, facilitates the adsorption of oxygen intermediate species at the catalytic active sites, which likely results in a higher surface coverage of these species and shifts the RDS toward an electron transfer-controlled process.^79,80^ All these findings confirm the superior catalytic activity of NSB-GNR over its double-doped counterparts. Additionally, NSB-GNR exhibited satisfactory stability, as shown in Figure 6d, retaining 67.9% of its initial current density after 85,000 s, highlighting its potential as a durable metal-free ORR electrocatalyst for alkaline electrochemical energy conversion and storage systems, such as fuel cells and metal–air batteries.

**Figure 6:**
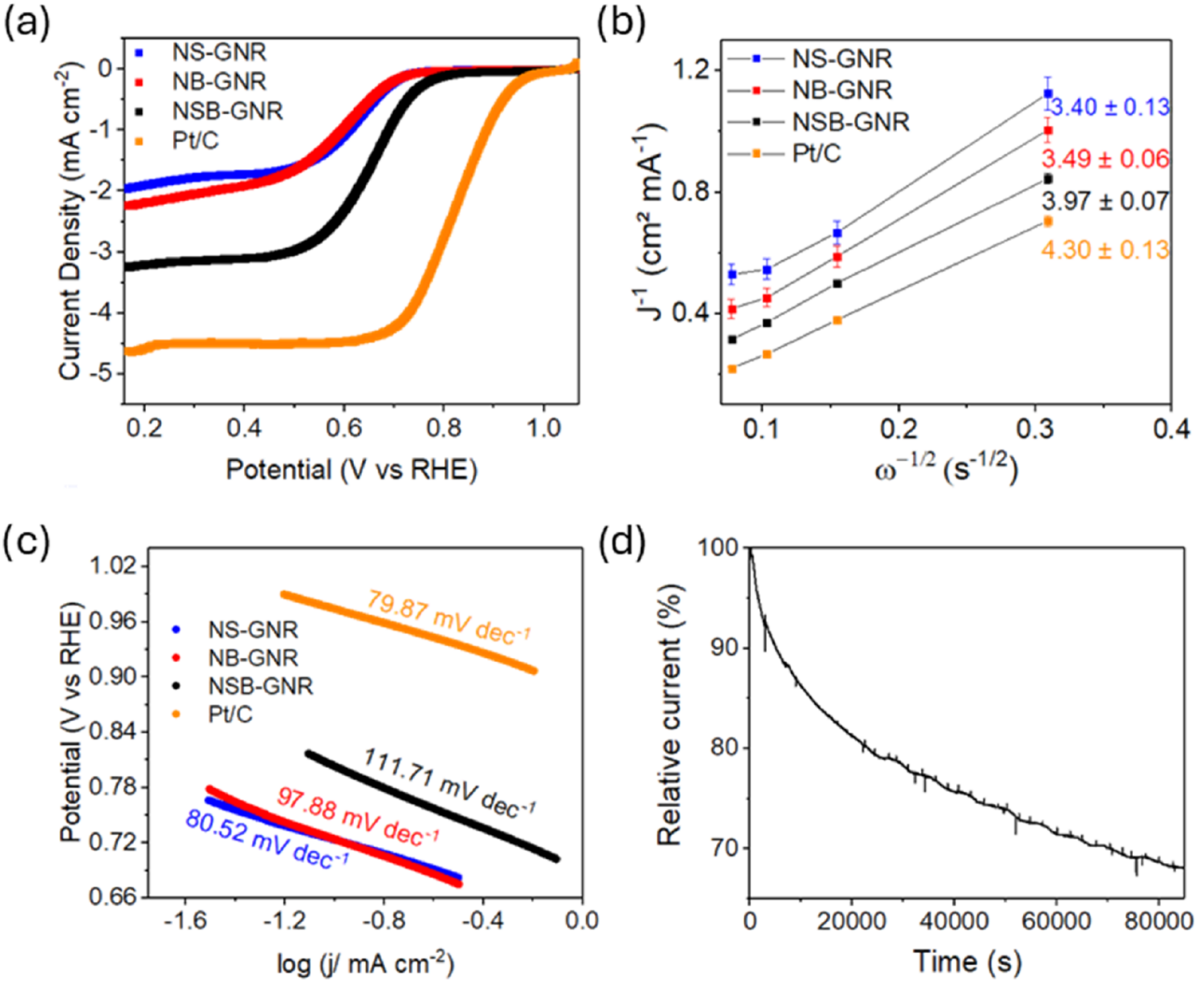
Electrochemical characterization of NSB-GNR, NB-GNR, NS-GNR, and Pt/C electrodes: (a) LSV curves recorded in oxygen-saturated 0.1 M potassium hydroxide at 1600 rpm; (b) K–L plots obtained from the average of three potentials (0.2, 0.3, and 0.4 V vs RHE) from LSV curves at different rotation speeds (see Figure S4), with the calculated electron transfer number for each electrode presented; (c) Tafel plots derived from the LSV curves in (a); (d) long-term stability test for the ORR for NSB-GNR at 0.714 V vs RHE in 1.0 M KOH.

**Table 4:** ORR Electrochemical Parameters for NSB-GNR, NB-GNR, NS-GNR, and Pt/C Catalysts Obtained from LSV Measurements in Oxygen-Saturated 0.1 M Potassium Hydroxide

sample	*E*_onset_ (V vs RHE)	*E*_1/2_ (V vs RHE)	*J*_L_ at 0.2 V (mA cm^–2^)
NSB-GNR	0.805	0.658	–3.24
NB-GNR	0.732	0.617	–2.54
NS-GNR	0.724	0.640	–1.96
Pt/C	0.975	0.808	–4.62

As mentioned previously, only a few studies have evaluated the combined effect of nitrogen, sulfur, and boron codoping. Chauhan et al.,^26^ through density functional theory, showed that the simultaneous incorporation of the three heteroatoms can modulate the adsorption strength and charge distribution on the catalyst surface. In their model, the H_2_O molecule was used as a representative probe for ORR, providing a simplified means to assess product–surface interactions and infer catalytic efficiency toward oxygen reduction.

Xiao et al.^27^ demonstrated that the codoping with nitrogen, sulfur, and boron induces complementary effects, where nitrogen and sulfur reduce the electron cloud density around carbon and oxygen atoms, while boron generates carbonaceous defects that increase the number of active sites. The incorporation of these elements also enlarged the specific surface area. In contrast, the findings in the current work reveal that boron addition reduces the surface area and has a minor influence on defect generation. These discrepancies are mainly attributed to the distinct synthesis route and the boron precursor employed. Specifically, Xiao et al.^27^ used sodium tetraborate decahydrate and a high-temperature thermal process, which not only facilitated higher boron incorporation but also prevented particle agglomeration, ultimately leading to a greater defect density and higher surface area. This difference indicates that the enhanced ORR performance of NSB carbon catalysts is driven primarily by the synergistic electronic effects of the heteroatoms rather than by changes in textural properties.

Liu et al.^25^ attributed the enhanced ORR performance of NSB codoped carbon nanotubes to the elimination of oxidized sulfur species (C–SO_*x*_–C) after boron incorporation. In this study, although Table 2 shows a slight decrease in oxidized sulfur species after boron incorporation, ICP-OES analysis revealed a higher total sulfur content in NSB-GNR compared with NS-GNR (Table 1). Hence, the removal of oxidized sulfur is unlikely to be the main reason for the observed catalytic enhancement. The superior ORR activity of NSB-GNR is attributed to cooperative electronic modulation induced by the coexistence of N, S, and B dopants. This modulation manifests experimentally as (1) a shift in ORR kinetics and the rate-determining step, (2) selective promotion of the four-electron pathway, and (3) enhanced activity despite reduced surface area and pore volume, indicating an electronic rather than textural origin.

Despite the remarkable catalytic activity of NSB-GNR, the benchmark Pt/C still delivers superior *E*_onset_, *E*_1/2_, and *J*_L_ values (Table 4 and Figure 6). Nevertheless, the substantially lower cost and superior scalability of metal-free carbon catalysts can offset this performance gap, making them a promising alternative for practical applications.

## 4 Conclusions

In summary, N, S, and B dual- and tridoped graphene nanoribbon (GNR) catalysts were hydrothermally synthesized, systematically characterized, and investigated as efficient metal-free electrocatalysts for the oxygen reduction reaction (ORR). The successful incorporation of nitrogen, sulfur, and boron into the GNR structure was confirmed by EELS and FTIR analyses. Complementary XPS and ICP-OES results revealed that boron is predominantly located within the inner layers of the GNR. The hydrothermal introduction of boron strongly influenced the textural properties of NSB-GNR. The reduced pH caused by boric acid partially neutralized the surface charge, leading to decreased BET surface area and pore volume. These results demonstrate that the enhanced ORR performance of NSB-GNR arises from cooperative electronic effects among N, S, and B dopants, rather than from surface area or porosity, providing experimental evidence of multiheteroatom synergy in graphene nanoribbons.

Overall, this work advances the understanding of multiheteroatom synergy in carbon frameworks and underscores the potential of low-temperature hydrothermal synthesis as a scalable and sustainable strategy for developing next-generation metal-free electrocatalysts for energy conversion and storage systems.

## Supplementary Material



## Data Availability

The data supporting this article have been included as part of the Supporting Information.

## Notes

XPS high-resolution spectrum for B 1s in NSB-GNR, XPS survey spectrum and high-resolution spectra for the NB-GNR catalyst, XPS survey spectrum and high-resolution spectra for the NS-GNR catalyst, and RDE polarization curves for ORR on different catalysts (Figures) (PDF)
