# Supplementary material for: Synergistic Electronic Modulation in Nitrogen, Sulfur, and Boron-Doped Graphene Nanoribbons for Enhanced Oxygen Reduction Electrocatalysis
Source: ACS Omega. 2026 Feb 24;11(9):15377–88. doi: 10.1021/acsomega.5c12983 (PMC12980185; doi:10.1021/acsomega.5c12983)
Supplement: Supplementary file 1 [file ao5c12983_si_001.pdf]

SUPPLEMENTARY MATERIAL

Synergistic electronic modulation in nitrogen, sulfur, and boron-doped graphene nanoribbons for enhanced oxygen reduction electrocatalysis

Giancarlo S. Dias[1,2], Matthew Labbe[2], Anqiang He[2], Richard Landers[3], Josiel M. Costa[4*], Ambrósio F. de Almeida Neto[1*], Douglas G. Ivey[2]

[1] *Departamento de Desenvolvimento de Processos e Produtos, Faculdade de Engenharia Química (FEQ), Universidade Estadual de Campinas (UNICAMP), Avenida Albert Einstein, 500, 13083-852 Campinas, São Paulo, Brazil*

[2] *Department of Chemical and Materials Engineering, University of Alberta, Edmonton, Alberta, Canada, T6G 1H9*

[3] *Departamento de Física Aplicada, Instituto de Física GLEB Wataghin (IFGW), Universidade Estadual de Campinas (UNICAMP), Rua Sérgio Buarque de Holanda, 777, 13083-859 Campinas, São Paulo, Brazil*

[4] *Faculdade de Engenharia de Alimentos (FEA), Universidade Estadual de Campinas (UNICAMP), Rua Monteiro Lobato, 80, 13083-862, Campinas, São Paulo, Brazil*

*corresponding author: josiel.martins.costa@gmail.com, ambreq@unicamp.br

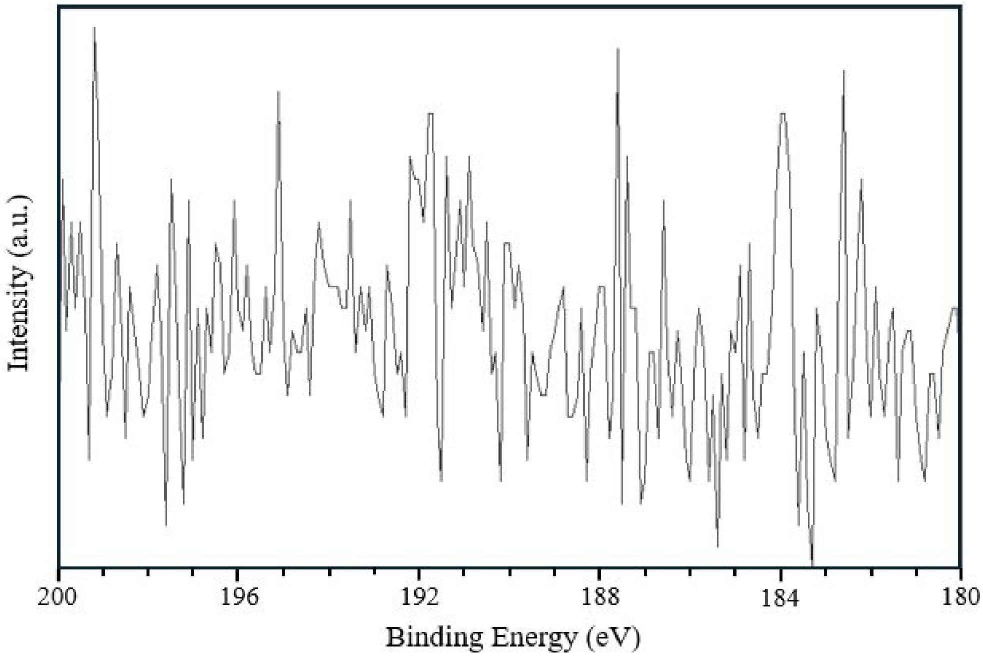

Figure S1. XPS high-resolution spectrum for B 1s in NSB-GNR.

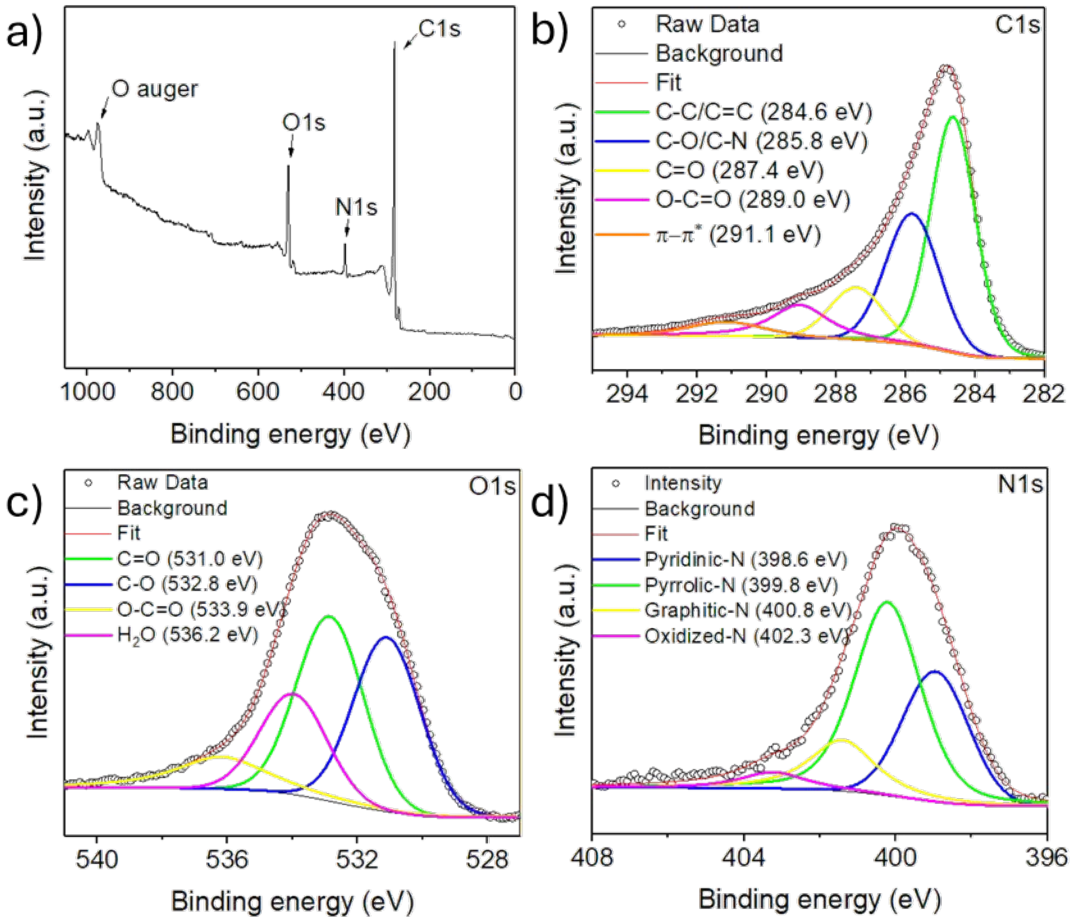

Figure S2. (a) XPS survey spectrum and high-resolution spectra for (b) C 1s, (c) O 1s, and (d) N 1s of the NB-GNR catalyst.

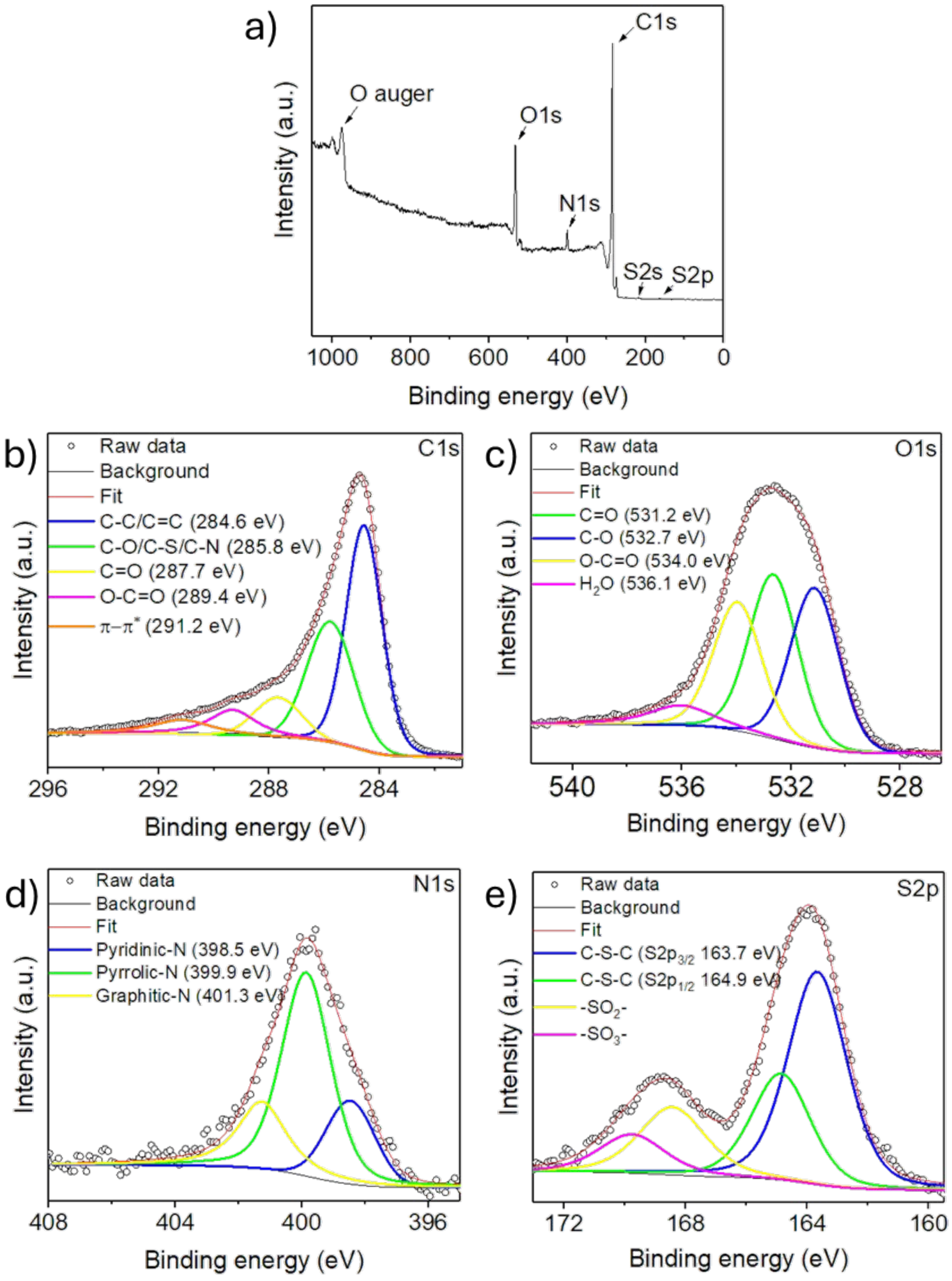

Figure S3. (a) XPS survey spectrum and high-resolution spectra for (b) C 1s, (c) O 1s, (d) N 1s, and (e) S 2p of the NS-GNR catalyst.

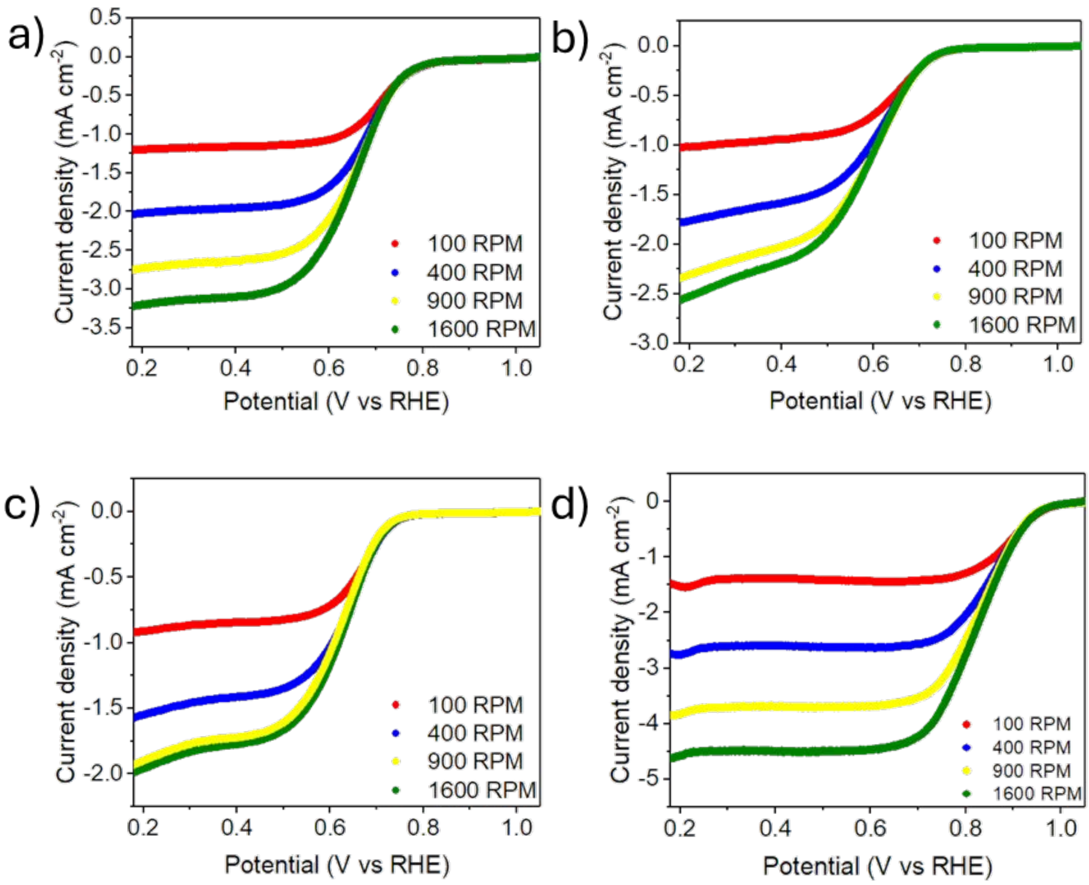

Figure S4. RDE polarization curves for ORR on (a) NSB-GNR, (b) NB-GNR, (c) NS-GNR, and (d) Pt/C at 100-1600 rpm in oxygen-saturated 0.1 M potassium hydroxide at 5 mV s^{-1}.
